# A framework for biomedical figure segmentation towards image-based document retrieval

**DOI:** 10.1186/1752-0509-7-S4-S8

**Published:** 2013-10-23

**Authors:** Luis D Lopez, Jingyi Yu, Cecilia Arighi, Catalina O Tudor, Manabu Torii, Hongzhan Huang, K Vijay-Shanker, Cathy Wu

**Affiliations:** 1 Department of Computer and Information Sciences, University of Delaware, Newark, DE, USA; 2 Center for Bioinformatics and Computational Biology, University of Delaware, Newark, DE, USA

## Abstract

The figures included in many of the biomedical publications play an important role in understanding the biological experiments and facts described within. Recent studies have shown that it is possible to integrate the information that is extracted from figures in classical document classification and retrieval tasks in order to improve their accuracy. One important observation about the figures included in biomedical publications is that they are often composed of multiple subfigures or panels, each describing different methodologies or results. The use of these multimodal figures is a common practice in bioscience, as experimental results are graphically validated via multiple methodologies or procedures. Thus, for a better use of multimodal figures in document classification or retrieval tasks, as well as for providing the evidence source for derived assertions, it is important to automatically segment multimodal figures into subfigures and panels. This is a challenging task, however, as different panels can contain similar objects (i.e., barcharts and linecharts) with multiple layouts. Also, certain types of biomedical figures are text-heavy (e.g., DNA sequences and protein sequences images) and they differ from traditional images. As a result, classical image segmentation techniques based on low-level image features, such as edges or color, are not directly applicable to robustly partition multimodal figures into single modal panels.

In this paper, we describe a robust solution for automatically identifying and segmenting unimodal panels from a multimodal figure. Our framework starts by robustly harvesting figure-caption pairs from biomedical articles. We base our approach on the observation that the document layout can be used to identify encoded figures and figure boundaries within PDF files. Taking into consideration the document layout allows us to correctly extract figures from the PDF document and associate their corresponding caption. We combine pixel-level representations of the extracted images with information gathered from their corresponding captions to estimate the number of panels in the figure. Thus, our approach simultaneously identifies the number of panels and the layout of figures.

In order to evaluate the approach described here, we applied our system on documents containing protein-protein interactions (PPIs) and compared the results against a gold standard that was annotated by biologists. Experimental results showed that our automatic figure segmentation approach surpasses pure caption-based and image-based approaches, achieving a 96.64% accuracy. To allow for efficient retrieval of information, as well as to provide the basis for integration into document classification and retrieval systems among other, we further developed a web-based interface that lets users easily retrieve panels containing the terms specified in the user queries.

## Introduction

With the emergence of high throughput techniques to study protein-protein interactions (PPIs) and protein complexes, such as two-hybrid system, mass spectrometry, fluorescence and protein chip technology, the number of known PPIs has increased exponentially in the last few years [1]. New findings are reported in scientific publications, or directly submitted to biomedical databases. For example, consider the MEDLINE database [2], maintained by the National Library of Medicine (NLM), and which is searched through the PubMed search engine. This collection of documents contains over 22 million biomedical literature citations and is growing at a rate of over 800, 000 articles per year. A naive search for nouns and verbs derived from the word "interaction", which might be hinting to the existence of a protein-protein interaction, returns around 1 million results containing these words in the title or the abstract of the MEDLINE citations. This number increases considerably when other query terms are used, which might be pointing to the existence of a protein-protein interaction, such as variations of the words "binding", "association", "dissociation", etc.

To access information about PPIs in the biomedical literature, life scientists spend increasingly more time searching online for the proteins of their interest. Some of the figures contained within the biomedical literature might play an important role in illustrating concepts, methodology, and results. However, figures are not commonly available in biomedical databases as stand alone entities that could be readily used by automated systems. Only a few databases require authors to submit figures and captions in separate files for easy access. Therefore, most of the figures are found embedded inside of the articles containing them, thus significantly reducing their automatic accessibility. In addition, a single figure may contain multiple sub-figures (panels), each of which corresponding to a different type of information.

Recent literature mining approaches, biomedical information retrieval systems, and document triage methods, have started using information from both the figures and the captions contained within scientific articles. For example, the National Library of Medicine (NLM) has developed several tools for content-based image retrieval. Notably, the system developed by Demner-Fushman *et al. *[3] uses figure captions to automatically classify and archive various types of CR/MRI images. This system helps retrieve images with relevant evidence in support of clinical decisions. Another example is the system developed by Stanley *et al. *[4] for retrieving X-Ray imagery to assist life scientists in identifying abnormal growth in cervical vertebrae. Similarly, Shatkay *et al. *[5] have developed a method that represents documents based on image panels and text, and they use this representation to classify biomedical documents. To do so, the authors obtain image panels from publications, classify the panels by type, cluster together panels that share similar image-features, and define each document by the image clusters identified within. In a similar vein, Murphy *et al. *[6] proposed a framework for extracting, segmenting, and classifying fluorescence microscope images to identify documents containing information relevant to protein subcellular localization. This framework can automate the collection, organization and summarization of biological data [7].

Based on the results of the above approaches, we can conclude that figures included in the biomedical literature, along with their associated captions, can effectively improve the performance of text retrieval and mining tools. The figures provide a unique source of information that typically complements the facts described in the text of the articles. This information is usually a more concise description of the ideas, experiments, and results detailed in the scientific publications, and it usually contains a high number of content-bearing words that effectively summarize the contributions of the article.

The task of accurately separating and then labeling each panel in a multimodel figure is a crucial task for content-based document classification and retrieval. This problem is particularly challenging for a number of reasons. First, different types of panels can still share similar image characteristics (i.e., barcharts and linecharts). Second, certain types of biomedical figures are text-heavy (e.g., DNA sequences and protein sequences images), thus varying from traditional images. As a result, classical image segmentation techniques based on low-level image features, such as edges or color, are not directly applicable to robustly partition multimodal figures into single modal panels.

In this paper, we present an approach for identifying and segmenting unimodal panels from a multimodal figure. Our approach actively integrates information extracted from captions, such as the number of panels in the figure and their description, with textual and pixel-level information extracted from the figure, to robustly partition the figure panels. Specifically, we first identify the label style (e.g., a, b, 1, 2) used to mark panels. Then, we partition the figure into a set of bounding boxes of connected components. We perform a lexical analysis on the text within the figure to identify the panel labels. Finally, we combine this information to determine the layout that optimally partitions the figure.

To evaluate the performance of our proposed solution, we focus on the literature containing protein-protein interactions (PPIs) as a use case [8]. Our dataset consist of 2, 256 full-text articles randomly selected from the annotated corpus provided by the MINT database [9]. These documents contain 13, 435 figure-caption pairs divided in 41, 341 panels. Our experimental results show that our segmentation approach greatly surpasses pure caption-based and pure image-based approaches, and it recovers 96.64% of the panel-subcaption pairs in the dataset. This manuscript is an expansion of the proceedings of the 2012 IEEE International Conference on Bioinformatics and Biomedicine (BIBM) $\tilde{\text{c}}\text{itelopezSegmentation}12$. This extended version gives a complete description of our system setup, models, and algorithms used in our solution. Additionally, the discussion presented provides a more detailed analysis of our experimental results, potential applications and future work.

## Methodology

**Figure 1:**
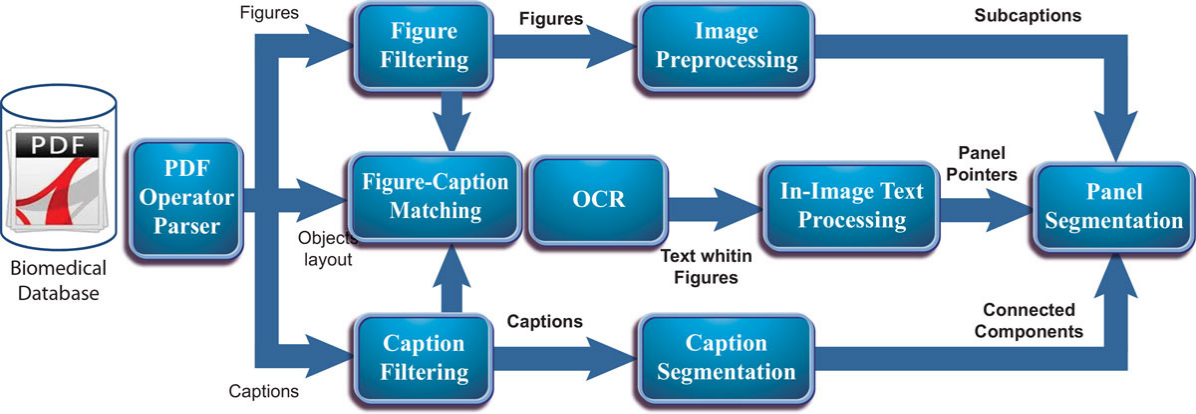
**Processing pipeline**. Pipeline of our proposed approach for automatically segmenting unimodal panels from full-text articles.

Our processing pipeline consists of eight major components (see Figure 1): a **PDF Operator Parser **that extracts layouts of figures and captions in a PDF document, a **Figure Filtering **module that removes journal logos incorrectly identified as figures, a **Caption Filtering **module that removes the captions incorrectly harvested by the operator parser, and a **Figure-Caption Matcher **that evaluates document layouts for accuracy in associating the recovered figures and captions.

Given the extracted figure-caption pairs, we first identify the number of panels from the caption and, separately, identify the number of panels from the image. The **Caption Segmentation **module performs a lexical analysis to identify the number of panels from the caption, and generates the corresponding subcaptions. Similarly, the **In-Image Text Processing **module identifies the corresponding number of panels in the image by performing a lexical analysis on the text within the figure. Additionally, an **Image Preprocessing **module performs a connected components analysis using pixel-level information to partition each figure into a set of bounding boxes. Finally, the **Panel Segmentation **module combines the results obtained from the previous three modules to first estimate the correct number of panels, and then to partition the figure using the image-level and label-level details.

### PDF Operator Parser

The PDF format stores a document as a set of operators that describe the graphical and textual objects to be displayed in the pages. Each PDF operator has specific parameters to define its formatting and layout [10]. To open a PDF file and extract its operators, our solution directly uses the Xpdf tool. We then perform further analysis on the layout information of these operators to identify and fix errors, and to improve the accuracy of associating figures with captions.

We recover layouts of captions and figures using an event-driven Finite State Machine (FSM) with four states: "**Reading Operator**", "**Reading Caption**", "**Reading Image**", and "**Finish FSM**".

The "Reading Operator" is the initial state. In this state, the FSM simply reads the operators extracted from the PDF file. To identify potential captions, we look at all the paragraphs starting with "Fig." or any textual variations of it ("FIG.", "Figure", etc.), which create a transition to the "Reading Caption" state. Since PDF files commonly stores operators containing a single line of text. The captions can be split across multiple consecutive textual operators. Therefore, the FSM stays in the "Reading Caption" state until it identifies the last operator that contains text from the current paragraph or when finish processing all the operators in the file. And then stores the caption by merging the string of text from the operators previously processed.

When a graphical operator is found in the "Reading Operator" state the FSM transits to the "Reading Image" state. In this state the FSM recovers the image and its layout (i.e. the position, size, and orientation of the figure in the page) and stores this information in a linked-list structure for easy access in further processing stages. Finally the "Reading Image" state returns control to to the initial state by defining a transition with a NULL event.

When the FSM finish processing all the operators in the PDF file, it creates a transition from either the "Reading Operator" or the "Reading Caption" state to the "Finish FSM" state where it ends the recovering process.

### Figure filtering

Biomedical full-text articles typically incorporate a significant number of non-informative figures (e.g., conference and journal logos). The inclusion of those figures, not only incurs additional computational processing cost but also makes it more difficult to match between figures and captions [11]. Therefore, our solution first analyzes the page and figure layouts to automatically identify and remove the non-informative figures. Specifically, we evaluate the position of each extracted figure with respect to a pre-computed rectangle bounding the articles text, and exclude the figures that are outside the rectangle.

**Figure 2:**
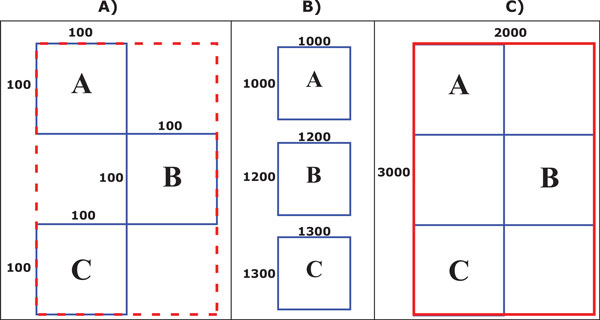
**Rendering images**. Rendering a high resolution figure from a set of subfigures. (A) Whole figure layout (consiting of three subfigures, denoted A, B and C, in the low resolution page space. (B) Actual subfigure dimensions. (C) Reconstructed high resolution figure.

The second obstacle we encounter is the lack of a standard for embedding figures in PDF files. In the ideal case, each figure in the article should correspond to a graphical operator in the PDF document. However, its a common practice to divide the figures in a set of disconnected subfigures, which do not necessarily correspond to the subfigures or panels that our approach is trying to identify. Our approach tries to group these subfigures into a single full-resolution figure, step which we call N-to-1 subfigure-figure correspondence. The detection of N-to-1 correspondence relies on the observation that, in a 1-to-1 correspondence, each operator defining a figure is followed by an operator defining a caption, whereas in the N-to-1 case, a set of consecutive operators defining figures would be followed by a single caption. Therefore, our solution groups the consecutive subfigures in a page preceding a caption. And then reconstruct the original figure from the set of subfigures. For this merging step, we compute the dimensions of the reconstructed figure and map the pixel data from each subfigure using their position and orientation in the document. Specifically, for each harvested subfigure, we obtain its scaling factor, by comparing its actual dimensions with its corresponding size on the reconstructed figure. The dimensions of the reconstructed figure are computed by applying the smallest scaling value, computed in the previous step, to the minimum bounding box surrounding the set of subfigures. We then map the pixel values of each input image to the final image using their position and orientation in the bounding box. This approach generates a high resolution image while avoiding undersampling. Figure 2 shows an example of the reconstruction process.

### Caption filter

**Figure 3:**
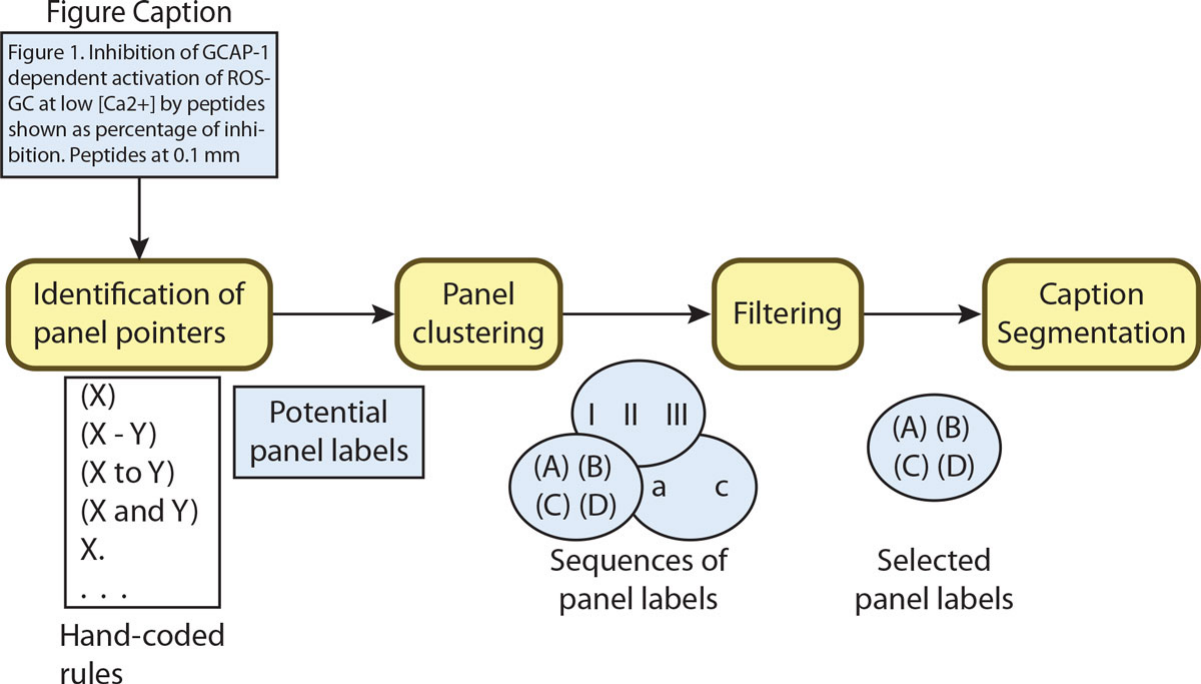
**Caption segmentation**. Overview of our approach to segment figure captions extracted from biomedical publications.

Although the FSM used in our approach correctly extracts the complete set of captions in the articles [11], it sometimes may incorrectly identify citations to figures as captions. Therefore, our solution uses the following approach to identify and remove the references to figures from the caption set: we first create a caption descriptor for every string of text in the set. Our solution uses only the first and second words in the caption. The proposed descriptor preserves the alphabetical characters (e.g A, a, B, b, etc), special characters (e.g. ')',']'), and punctuations (e.g '.' ',') from both words. We then substitute the numbers, typically in the second word, by the symbol *'*#*'*. Strings with identical descriptors are then clustered together, and the number of unique figures in each group with identical descriptor are then calculated by sorting them using the number of the figure they are referring. Finally, the group with the maximum number of unique links to figures is selected as the correct number of captions and the rest are simply discarded. This process if graphically shown in Figure 3.

### Figure-caption matcher

Once our method extracts the correct set of figures and captions, we link each figure to a caption. To perform this association, we developed an iterative matching technique that estimates the quality of the match between figures and captions occurring on the same page, by using both geometric and structural cues. Our method looks for three possible scenarios: 1) a 1-to-1 matching corresponding to 1 figure and 1 caption in a page, 2) an N-to-N matching corresponding to *N *figures and *N *captions in a page, and 3) an N-to-M matching corresponding to *N *figures and *M *captions in a page.

Given the extracted figures and captions, we first assign them a label corresponding to their structural position (e.g., left column, right column, and double column). For each non-empty page, we precompute the cost to associate all figure-caption pairs across the page. For simplicity, we use *f_i _*to denote the *i^th ^*figure, and *c_j _*to represent the *j^th ^*caption in the page. Our goal is to use geometric and structural attributes to compute the optimum match between the corresponding objects.

For a given figure-caption pair *f_i _*and *c_j _*we compute their matching cost *C*(*f_i_*, *c_j_*) as the minimum distance $d_{f_{i},c_{j}}$ between their corresponding boundaries in the Euclidean space, multiplied by a penalty cost $p_{f_{i},c_{j}}$ that prioritizes the association between objects with similar layout: $C\left(f_{i},c_{j}\right)=d_{f_{i},c_{j}}*p_{f_{i},c_{j}}$. If *f_i _*and *c_j _*lay in the same column, their penalty is set to 1; otherwise it is set to an arbitrary high number, 10 in our experiments.

We use the previously constructed cost matrix to find the optimum match between the objects in each non-empty page. In the base case of a **1-to-1 matching**, we simply associate the one figure and the one caption found on that page. For more complex cases (e.g., **N-to-N matching **and **N-to-M matching**), we first apply a greedy approach to find the optimal association of the objects in a page. We repeat this process taking into consideration the un-associated objects from other pages as well. The steps in detail are as follow: (1) we associate the figure-caption pair that correspond to the entry having the minimum value in the matching cost table; (2) we recalculate the cost matrix excluding the associated objects from step 1; (3) we repeat steps 1 and 2 until we exhaust the figures, the captions, or both, from the current page; (4) we repeat the same steps on all the pages of the document; (5) the unmatched objects from all the pages are then included in a new cost matrix, where the matching cost will also include the disparity between their pages: $C\left(f_{i},c_{j}\right)=d_{f_{i},c_{j}}*p_{f_{i},c_{j}}*disparity_{f_{i},c_{j}}$.

### OCR

Similar to Xu *et al. *[12] and Kim *et al. *[13], we extract text embedded inside figures for indexing purposes. To do so, we use the commercial software ABBYY OCR ^©^, which simultaneously recognizes the textual parts and their spatial coordinates in the high resolution figure. To recover the text from fragmented figures, we simply parse the PDF file to identify all the strings of text that lie inside the bounding box of the reconstructed figure. The OCR software achieves high precision when recognizing English characters in the high resolution images. However, it performance is lower when extracting Greek and other special symbols [11].

### Caption segmentation

Previous figure extraction and figure-based document classification approaches largely disregarded using the information found within captions. Besides containing important semantic information about the facts stated in the paper, the captions can also reveal information that is important for the extraction of the figures, such as their number of panels. However, parsing the captions and dividing them into corresponding panel subcaptions can be particularly challenging, since there is no standard convention of how to mark the boundaries between subcaptions. Subcaption labels can be written using complex structures [14,15]. Furthermore, captions may also contain abbreviations, special characters, or ambiguous words that can be mistakenly identified as panel labels [15].

In our approach, we look at a caption to identify the number of panels that might be hinted for the associated image. Moreover, we break the caption into subcaptions, each corresponding to a description of a specific panel within the figure.

**Figure 4:**
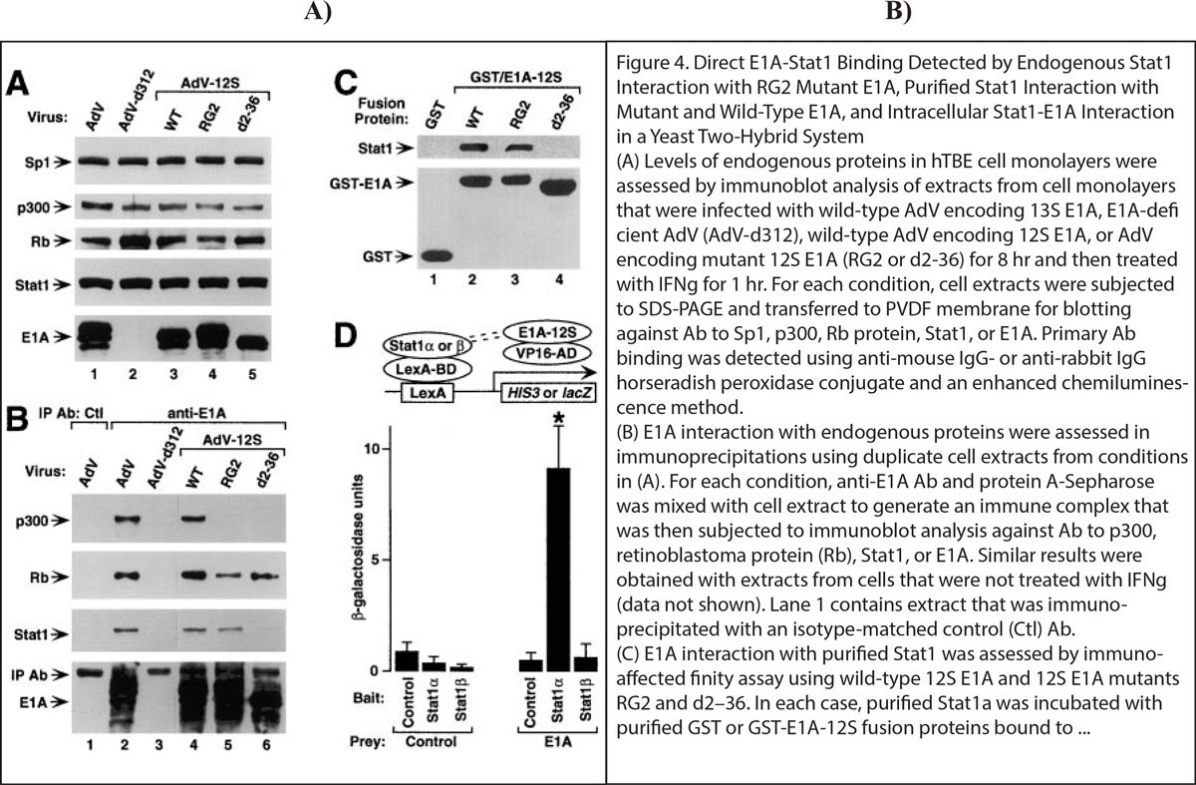
**Extraction example**. Example of a multimodal panels extracted from PMID9881977 containing two experimental methods for predicting and confirming a protein-protein interaction.

The first phase consists of a lexical analysis on the captions to identify a potential set of panel labels. Similar to Cohen *et al *[15], our solution uses a set of hand-coded rules to identify: 1) parenthesized expressions with a single label "(X)", "(x)"; 2) parenthesized expressions with a range of labels "(X - Y)", "(x - y)", "(x to y)", "(X, Y, and Z)", "(x and y)", etc; and 3) labels without parenthesis "X.", "x)", "X,", "x;". However, unlike [15], our solution performs an additional phase, described next, to filter out false positive labels. Our proposed method can recognize panel pointers with Roman and English characters, as well as with numbers. In Figure 4, we expect to identify four panel labels (e.g., "*(A)*", "*(B)*", "*(C)*", "*(D)*") in the caption extracted from *PMID9881977*.

The second phase analyzes the set of panel labels suggested by the first phase, in order to identify and subsequently remove the incorrect labels. We first cluster the panel labels into groups of similar types: English letters, Roman numbers, or Arabic numbers. The labels of the same type, which do not form a sequence, are then removed, as these could be errors introduced by abbreviations. If we encounter multiple occurrences of the same label, we simply keep the first occurrence and remove the rest. In the rare case when a single caption contains multiple sequences of the same type, we select the group with the highest cardinality and discard the rest. Finally, among the sequences that are left, we give priority to letters, then Roman numbers, then Arabic numbers.

In the last phase, we fragment the figure captions into subcaptions, using the position of the tokens identified as panel labels. For tokens containing a range of labels (e.g., (A-C)) we duplicate the subcaption as many times as the number of labels in the range (e.g., A-C will create three duplicated subcaptions, for A, for B, and for C).

### Image preprocessing

We use pixel level features (e.g., intensity values) to separate the objects from the background image (e.g., empty regions). We perform a connected component analysis on the resulting binary image, and then represent the objects in the figure as a bounding box.

Previous approaches [16-20] have shown that automatically separating the objects from the background is challenging, one of the main reasons being that biomedical figures typically contain a dense set of objects for which the intensity values may differ drastically. Therefore, in many cases, a single threshold value may not be enough to recover the correct set of objects in the figure. Other approaches minimized this problem by defining heuristics using domain-specific knowledge about the images. Specifically, Murphy *et al. *[16] identified discontinuities in the intensity value (e.g. edges) and used this information to create horizontal and vertical cuts. Several approaches utilized the Otsu filter [21] to compute the optimum value that minimizes the intraclass variance in the figure. The figures are then fragmented using the regions with the minimum variance [17,18].

In our approach, we compute the intensity value that best separates the foreground region using the triangle method first described in [22]. To briefly summarize, this method starts by constructing the histogram of the pixel intensity values. Next, a line is created by connecting the minimum non-zero value with the highest cumulative value in the histogram. We then measure the segmentation quality of each histogram bar lying inside the line intervals by computing the distance from the precomputed line to the peak of each bin. Finally we choose the bin with the maximum distance as the optimum threshold value. This technique has been widely used in the biomedical domain to effectively segment TEM images [23], microscopic blood images [24], and noisy images with low contrast [25].

**Figure 5:**
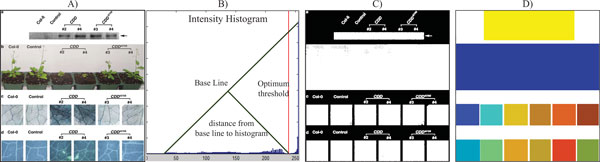
**Image processing**. A sample figure extracted from PMID16003391 describing our image processing algorithm. (A) Original image; (B) Optimum threshold value computed using the triangle method developed by Zack et al. [22]. (C) Binarization result using the threshold value calculated by the triangle method [22]; (D) Minimum area rectangles bounding the connected components in the figure.

The final result is achieved by removing the regions in the segmented image identified as text by the OCR software, and then creating a set of connected components (CC) [26] from the resulting figure by combining all the foreground pixels using a 4-neighbor connective scheme. To improve robustness, we remove the regions whose area is smaller than a predefined threshold, 50 in our experiments, and then compute a minimum area box surrounding each of the remaining regions. Finally, to minimize the errors in the segmentation process, we merge disconnected regions by combining bounding boxes that overlaps. This process is graphically shown in Figure 5.

### In-image text processing

We perform a lexical analysis on the text extracted from the OCR software to identify the set of potential panel labels. Although we expect the outcome to be the same, this step is different from the caption analysis module. The use of OCR systems to identify text from biomedical figures can potentially generate multiple additional problems (e.g., most OCR systems identify random letters in barcharts and figures with complex backgrounds, and fail to recognize letters embedded in low contrast regions). Thus, our system can incorrectly identify these noisy characters as protein/gene names or panel labels. In addition, the set of panel labels can contain gaps that corresponds to unrecognized characters [13].

**Figure 6:**
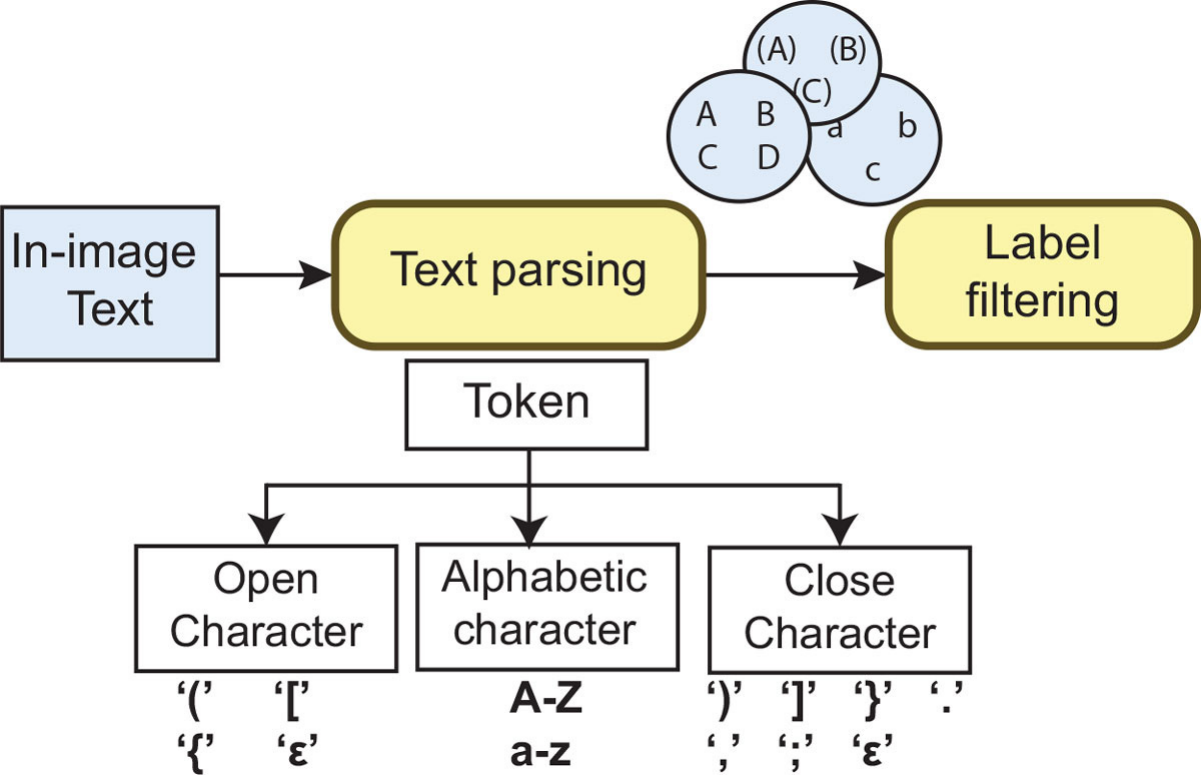
**Text processing**. OCR-based approach to identify panel pointers from biomedical figures

As Shown in Figure 6, our approach resolves these problems by first using a parse tree to recognize the panel label from the text recognized by the OCR software (e.g., A), B. (C), D, etc). Figure 6 shows a simplified view of the parse tree used to identify tokens from the text within the figures. Each extracted token represents a potential label of a panel (subfigure). We roughly classify the set of panel labels identified by our approach in three categories. 1) **simple labels **that consist of single characters (e.g., A, B). 2) **right-closed labels **that consist of a letter followed by a right delimiter, such as parentheses, brackets, or punctuation sings. And 3) **closed labels **that consist of an alphabetic character surrounded by delimiters (e.g., "(A)", "[B]").

Similar to the caption analysis module, once we finish recovering the set of panel labels from the image text, we cluster them into groups of similar types (e.g., {"a,","b,","c,"}, {"I","II","III"}). Each label in the non-empty sets is then sorted using their alphabetical position (e.g. position('b') = 2, position('D') = 4). In theory, our approach should recover only one group containing the real set of panel labels. In reality, it may also generate incorrect groups from noisy characters. To make sure that the mistakes are eliminated, we assess them to compute their confidence, we then select the group with the highest confidence score and discard the other groups. Specifically we define our heuristic to assess the confidence of set *i *as $confidence_{i}=\frac{1}{1+Gaps}*Panels$. Where *Panels *is the total number of panels identified in the figure, and *Gaps *is the total number of gaps in the sequence.

### Panel segmentation

**Figure 7:**
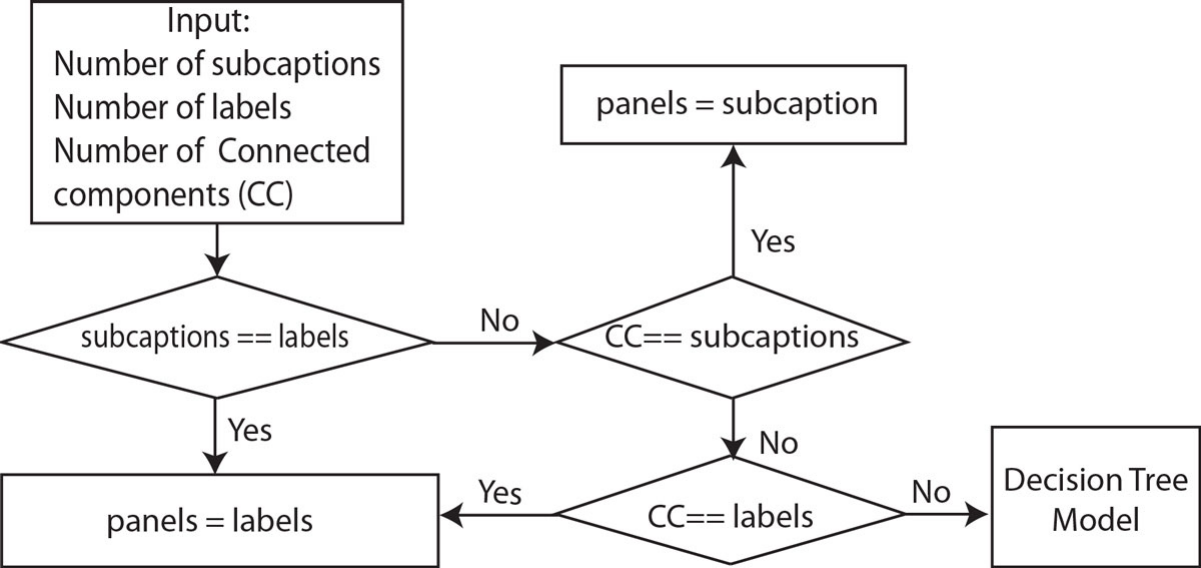
**Panel estimation**. Algorithm flow for estimating the number of panels in a multimodal biomedical figure.

The last step consists of segmenting the figure into panels using the information extracted from the previous four modules. To do so, we utilize the number of labels, connected components, and subcaptions obtained in the previous modules to estimate the correct number of panels in the figure. The flowchart of our algorithm is graphically shown in Figure 7. Notice that since the number of labels and CC were recovered from noisy data, they may differ from the real set of panels. Therefore, we first evaluate the subcaptions, which is our most confident set, with the reported number of panel labels. If this values are consistent we simply use it as the estimated number of panels. Otherwise, if their values are different (e.g., one or both approaches failed to recover the correct number of panels in the figure) we use the number of CC to determine the set that contains the correct number of panels.

**Figure 8:**
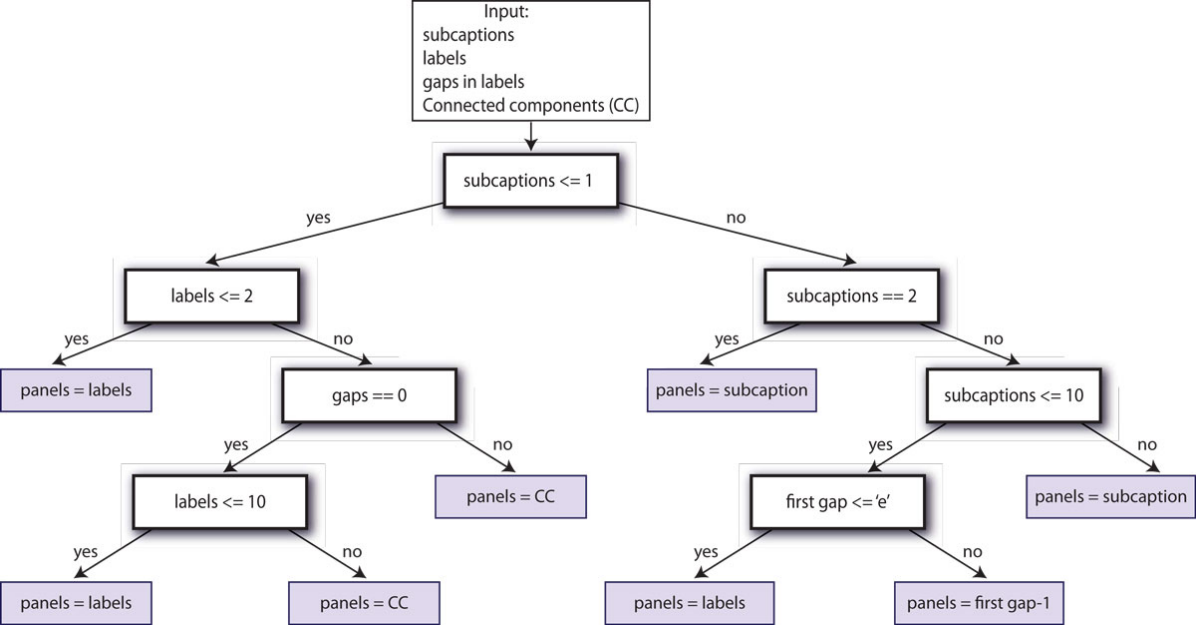
**Decision tree**. Decision tree trained to estimate the number of panels in a figure.

In the rare case when our approach fails to identify the correct number of panels from the recovered CC, labels, and subcaptions sets (e.g., the sets have different cardinalities), we estimate the number of panels using a decision tree algorithm C4.5 [27,28]. We trained our decision tree model using 200 figures randomly distributed in our proposed image taxonomy, these images were excluded in the overall evaluation of our system. We manually annotated the training images to identified their correct number of figure-captions pairs ((see Figure 8)). We trained our decision tree model by directly using the cardinality of the panel labels, sub-captions, and connected components as features. We included the number of gaps used to estimate the confidence of the sub-captions as an additional feature. The proposed decision tree model generates a value that we used as the correct number of subfigures in further processes.

**Figure 9:**
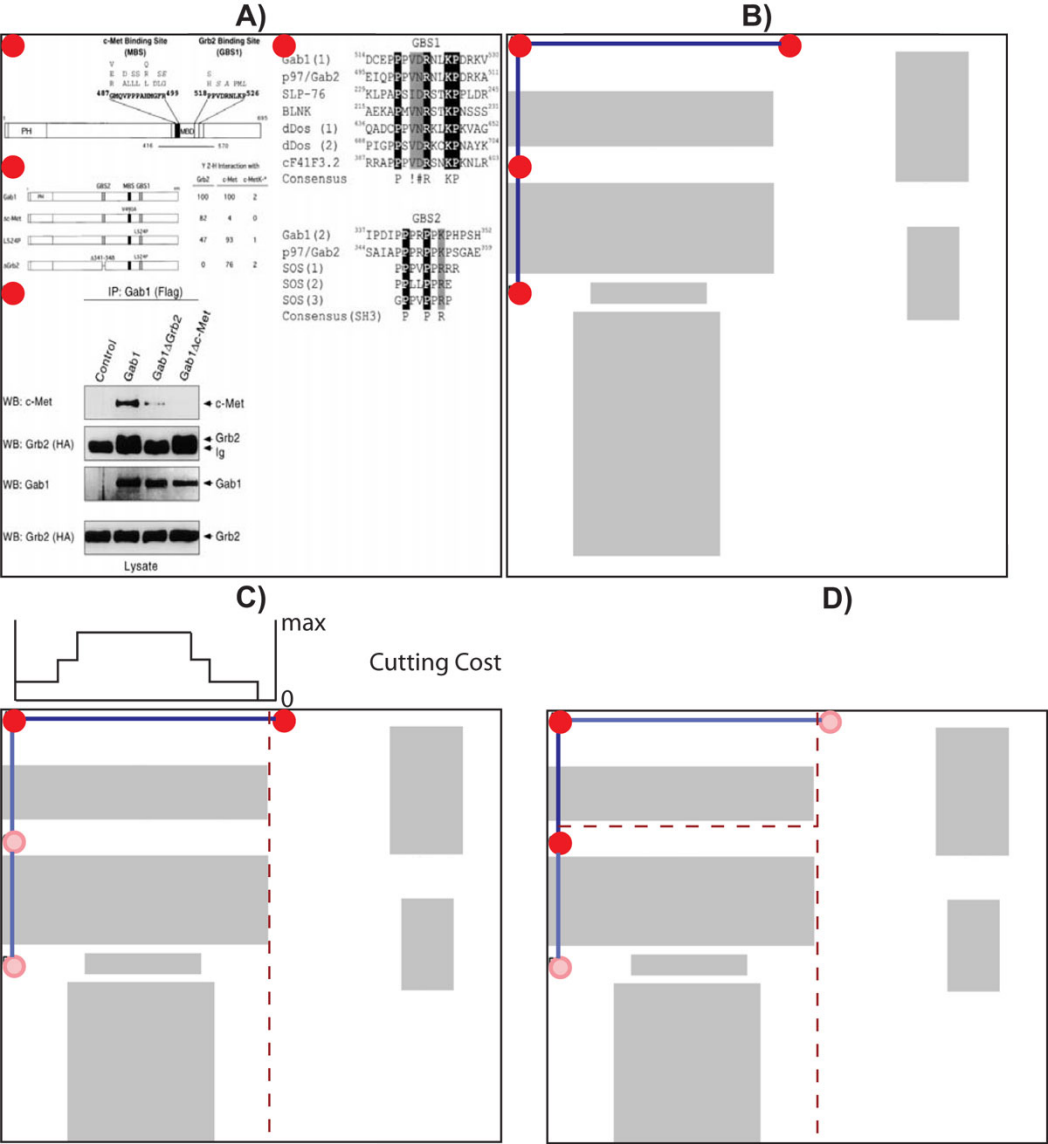
**Panel partitioning**. Partitioning the figures using geometric clues. (A) a sample input image extracted from PMID10871282 (he panel labels are highlighted in red circles); (B) shows the constructed layout graph; (C) shows the computed set of costs to perform a vertical cut in the current edge (the partition is placed in the zone with minimum cost); (D) shows the final image layout after evaluating the intersection of two optima partitions.

**Figure 10:**
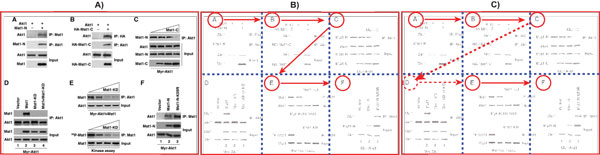
**Estimating missing panel Label**. Estimating the position of unrecognized pointers. (A) Sample input image extracted from PMID17932490; (B) Layout graph containing a gap in the first column; (C) Our final recovered layout graph including the estimated node "D".

We use the position of the panel labels to estimate the figure layout. Specifically, we start by constructing a layout graph *G *= (*V*, *E*), where each node *v_i _*∈ *V *corresponds to the position of a recovered panel label in the figure (Figure 9A), and the edges *E *connect each node to its closest horizontal and vertical neighbors (Figure 9B). Next, given a pair of adjacent nodes, *v_i_*, *v_j _*∈ *V*, that are connected by an edge *v_k _*∈ *V*, we compute the cost of cutting the figure for each pixel along the edge *v_k _*(Figure 9C). To minimize potential segmentation errors and reduce the time required to fragment the figure, we directly use the dimensions of the connected components to compute the cutting cost. Specifically, for every pixel (*x*, *y*) that lies on an edge (*i*, *j*) we project a line perpendicular to the edge direction that intersects the image coordinate (*x*, *y*), and find the connected components that intersects the projected line. The total cost of cutting the figure in pixel (*x*, *y*) is defined as the sum of the dimensions of the intersected connected components. We simply select the line with the minimum cost as the optimum position to partition the figure. When two optimum partitions (horizontal and vertical) intersect (Figure 9D), we evaluate their cost and stop the partition with a higher value at the intersection point. Figure 10 shows the pseudo code of our image segmentation algorithm.

**Figure 11:**
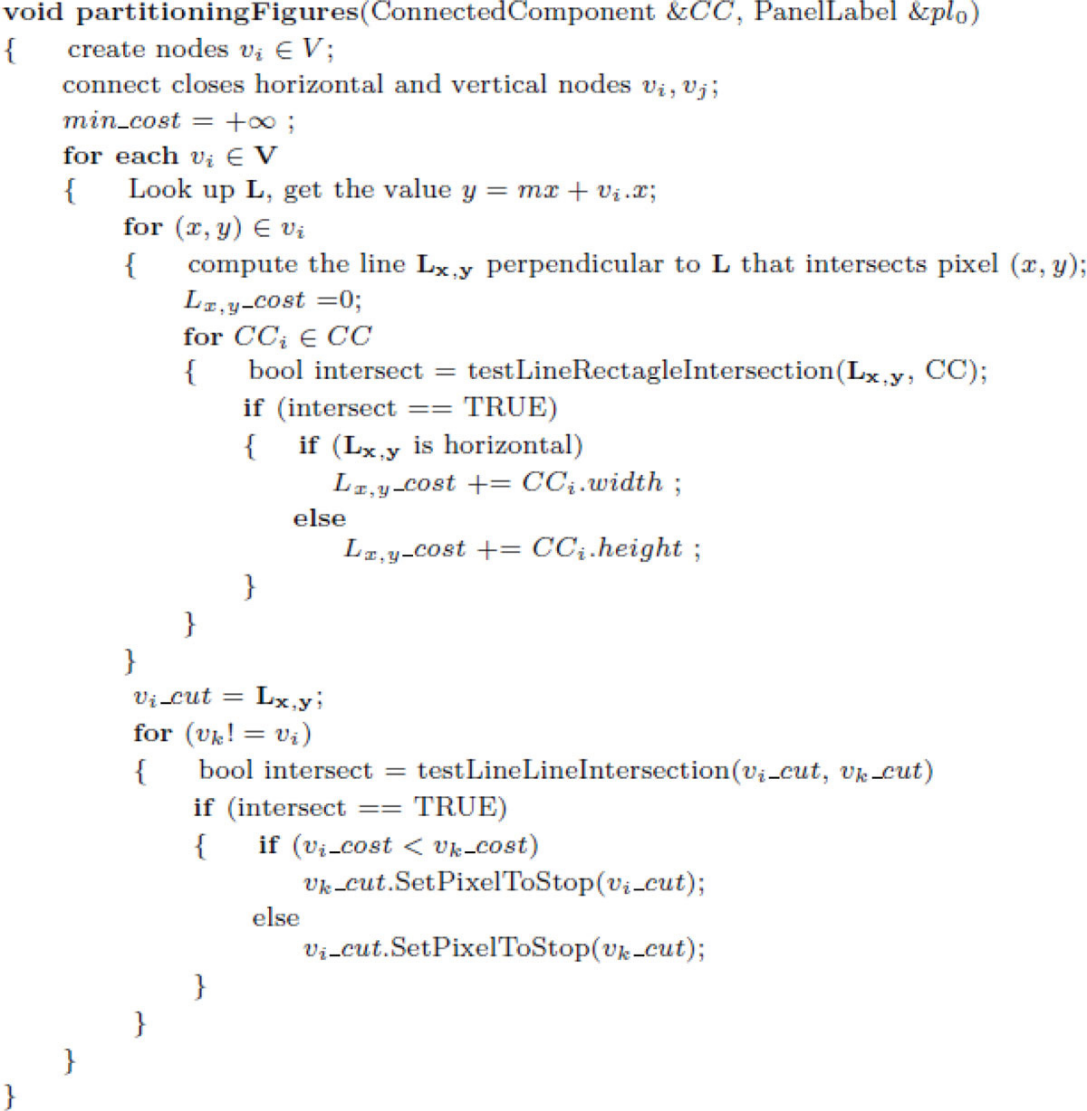
**Figure segmentation**. Pseudo-code of our figure segmentation algorithm.

Ideally the set of panel labels extracted from the text embedded in the figure should contain the complete set of panel labels in the figure. However, a common problem with current OCR engines, both commercial and open source, is that they are trained to recognize letters with good contrast and low noise levels, and they typically achieve a low performance (e.g. inaccurate or unrecognized characters) when are used to identify text embedded in figures with complex texture patterns in the background. Thus, some characters in the set of panel labels can be potentially missed by the OCR engines (e.g., A, B, and D are detected, but C is not detected). Our approach minimizes this problem by creating special labels to fill the gaps in the sequence, and estimating their location in the figure. Specifically, we discretize the figure in a set of blocks, where each block contains a panel label. We then insert the missing gaps by analyzing the patterns in the resulting blocks. If the set of blocks consist of a single row or column we simply insert the missing panel pointer in the midpoint between the two adjacent panel pointers. For more complex cases (e.g., two dimensional block patterns), we estimate the position of the missing panel by following the sequence of the panels in the previous row or column, if the estimated block is empty we reserve this space for the new label. This process is demonstrated graphically in Figure 11. In this figure harvested from paper PMID16159897 [29] our approach failed to recognize the pointer to panel "D". Therefore, we estimate its position by using the edge between nodes "A" and "B", as well as the edge between nodes "C" and "E" to estimate the horizontal and vertical position respectively. Once we finish recovering the set of panel pointers, we construct a graph by connecting the closest horizontal and vertical nodes. We the partition the figure into panels by creating vertical cut for each horizontal edge, and a horizontal cut for each vertical edge.

## Results and discussion

### Data and gold standard

To test our proposed approach, we selected a set of 2, 035 PMIDs from the MINT dataset [9]. The MINT database contains manually curated articles with textual and graphical descriptions of Protein-Protein interactions (PPI), discovered using a variety of experimental techniques (e.g., Co-Immunoprecipitation, yeast two-hybrid, cosedimentation, and fluorescence technologies). We chose to use this dataset because we are particularly interested in extracting figures related to PPI assertions, and also the accompanying textual description of the methods, which is usually found within subcaptions or text embedded in panels. Since MINT does not store full-text articles, we manually download the selected articles from PubMed Central (PMC) or the publishers website.

Manually extracting figure-caption pairs for the creation of our ground truth data for 2, 035 articles is tedious and time consuming. Instead, we generated our gold standard set by first automatically extracting the panels and their subcaptions. A group of three annotators confirmed the correctness of the extracted data through a web interface. This process significantly reduced the effort and time to generate our ground truth data, which consists of 13, 147 figure-caption pairs containing 41, 341 panels with associated subcaptions. Three annotators completed the annotation of the figure-caption pairs in 180 hours.

### Figure-caption matching

An important feature of our system is that it can automatically remove journal logos. In our experiments, 4, 974 figures were correctly identified as logos. Notably, no logos occurring in the documents were missed by our system. For the task of merging subfigures into figures, our system identified 51, 247 subfigures, and used them to correctly recover 3, 341 figures of the total set of 3, 383 fragmented figures in the dataset.

**Figure 12:**
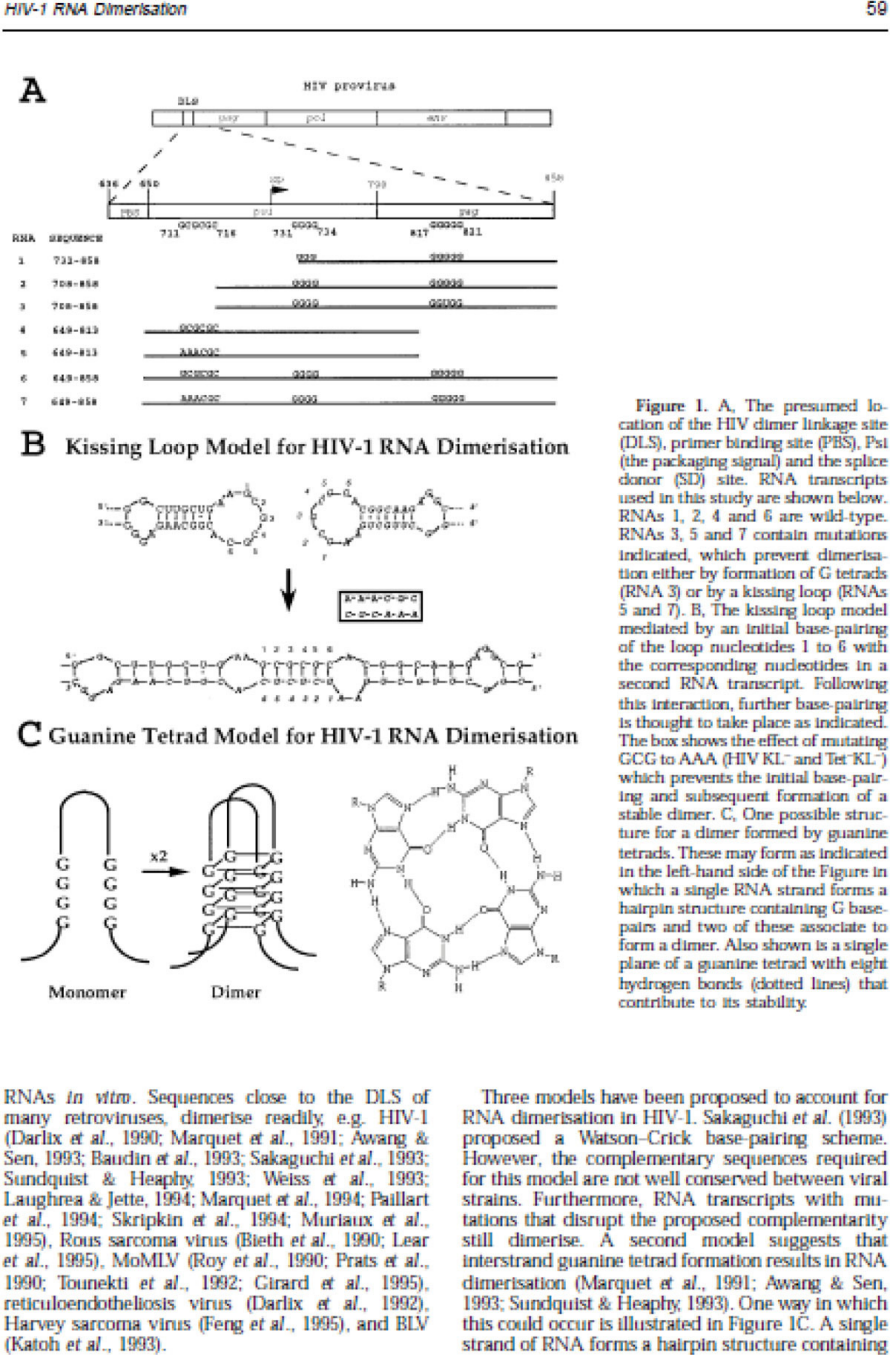
**Example of unrecognized image**. A sample figure from PMID8648648 [33] consisting of graphic primitives that our algorithm fails to extract.

To evaluate the performance of our figure-caption matcher, we applied the semi-automatic approach described earlier to obtain the ground truth figure-caption pairs for the documents. Our system correctly produced 97.85% of the figure-caption pairs. Figure 12 shows a gallery of figure-caption pairs harvested from article PMID16388599 [30]. We wanted to compare our system with the widely used free software *Some Pdf Images Extract*. Notably, SomePdf does not support extraction of figure captions. This is often the case for existing figure extraction tools, public or commercial, and we were unable to use them for our knowledge discovery purpose. The few systems that do support figure-caption extraction, such as [3,16], are not publicly available for comparison. Although using a different class of documents, both systems have reported that about 20% figures cannot be correctly extracted, whereas our system is able to consistently extract nearly all figures and their captions.

### Panel labels identification

Table 1 shows the performance of our system for the subtask of detecting panel labels within the figure. Our approach was able to identify the panel labels with a precision of 73.69% (9, 688 figures). Of these, 532 (4.04%) figures contain label gaps that are successfully fixed using our approach.

**Table 1:** Panel label segmentation results

Description		Images	Rate
Successfully Identified		9, 688	73.64%

Sequences with Gaps		532	4.04%

Cause of error	False Positive Labels	1, 479	11.24%
	
	Incomplete Set of Labels	1, 980	15.06%

Performance of our proposed solution for the subtask of detecting panel labels within the figure.

In order to determine the different sources of errors, we analyzed the remaining 3, 459 (26.31%) figures for which our system identified an incorrect number of panel labels. We found that for 1, 479 (11.24%) figures, the system reported more labels than the actual number of panels, thus making it impossible to divide the figure into more panels than it had. The main reasons for finding extra panel labels embedded in the figures are: (1) characters in zones with complex textures that cannot be recognized by the OCR engine; and (2) letters inside bar charts. We also found that 1, 980 (15.06%) figures contained an incomplete set of textual labels, and our system failed to fill in the gaps. We attribute three quarters of the errors to the OCR engine, which cannot determine the correct text in low contrast areas, or which erroneously identifies a letter as being another letter (e.g., "D" recognized as "I"). The other quarter we attribute to our system, for choosing the incorrect sequence of panel labels.

### Caption segmentation

Table 2 shows the performance of our system on the subtask of segmenting captions. In our evaluation, a caption is viewed as successfully segmented when it is fragmented in the same number of panels as provided by the gold standard for its corresponding figure. This does not necessarily mean that, when unsuccessful, our system made an error. Sometimes it happens that the caption gives no hint as to how many panels there might be in a figure.

**Table 2:** Caption segmentation results

Description		Images	Rate
Successfully Segmented		11, 984	91.15%

Cause of error	Incomplete Captions	673	5.12%
	
	Inconsistent Captions	292	2.22%
	
	Missing Identifiers	198	1.51%

Performance of our proposed solution for the subtask of segmenting subcaptions.

**Figure 13:**
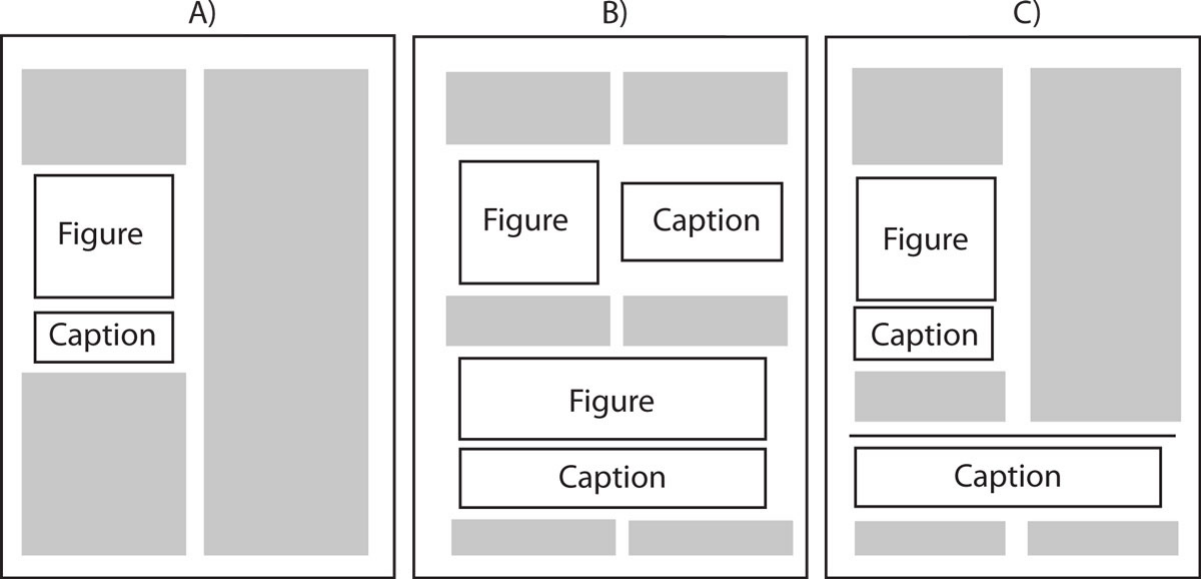
**Gallery of recovered panels**. A gallery of panel-subcaption pairs extracted by our framework. (A) The input image; (B) The segmented image panels with their corresponding subcaptions.

Given this definition of success, our caption parsing scheme is able to achieve an accuracy of 91.15%. In fact, there are 1.51% additional captions with no labels (just plain text) that describe a figure with multiple panels. In these situations, it would be impossible for our system to produce subcaptions. Figure 13, column B) shows a caption extracted from PMID16137684, accompanying a figure with multiple panels. This caption contains no labels to separate it into subcaptions. Figure 13, column B) shows a caption extracted from PMID15728366 that is composed of multiple separated columns of text. The separation of the caption in multiple groups of text in the article makes it impossible for our solution to determine the correct number of panels in the image.

We attribute 5.12% of erroneously segmented captions to the module that extracts figure-caption pairs. Biomedical figures tend to be highly complex, and consequently, they require long captions to describe them. As a result, it is a common practice to split the captions on different pages or to use complex document layouts to optimize the space, thus resulting in incomplete captions for a specific figure. The rest of 2.21% failing cases correspond to figures with inconsistent captions. For example, there are some captions that contain multiple non-overlapping sequences of subcaptions (e.g., A, B, C and a, b, c). It is not always clear which sequence to choose as the main one, especially if their cardinalities are the same. There are also captions mentioning protein names or abbreviations that are usually used to delimit a subcaption (e.g., (C) for carbon).

### Extraction of unimodal panels

Unlike previous approaches, our solution combines independent information from multiple modules to more accurately estimate the number of panels in a figure. Specifically, our solution identified the correct number of panels in 11, 973 (91.07%) cases from the total set of 13, 147 figure-caption pairs in our database. In particular, we identified the correct number of panels in 9, 294 cases by achieving a consensus in the sets of panel labels, sub-captions, and connected components. The number of panels in the remaining set of 2, 679 figures we correctly estimated using the decision tree model. Our approach achieved an incorrect consensus in 191 cases, and in 983 cases our decision tree model failed to estimate the correct number of panels in the figure.

The performance of the panel segmentation algorithm is calculated by looking at the various subpanels and subcaptions created and comparing them to the gold standard. Out of 41, 341 panels in the gold standard set, our system correctly segmented 39, 951 (96.64%).

Notice that the number of panels correctly produced by our approach is higher than the number of panels correctly estimated in the figures. This is because since our solution utilizes only local information to segment the panels, we are still able to produce a subset of correct panels even when our solution estimated an incorrect number of panels in the figure. Figure 13 shows an example where our segmentation approach fails. The caption in the article is separated in three independent columns of text, making impossible for our solution to extract the complete caption. A typical segmentation result generated by our approach is shown in Figure 12. Figure 12B shows the sets produced by our approach from the sample figures in 12A. The performance our our segmentation system is summarize in Table 3.

**Table 3:** Computational results

Description		Panels	Rate
Successfully Segmented		39, 951	96.64%

Cause of error	Incorrect Estimation of Panels	853	61.36%
	
	Incorrect Estimation of CC	253	18.20%
	
	Region Associated to Incorrect Panel	284	20.44%

Performance of our system for the subtask of segmenting unimodal panels from multimodal figures.

## Conclusions and future work

We have developed a comprehensive system for automatically extracting unimodal panels (subfigures) from full-text articles. Our hybrid framework was developed based on the observation that the text from captions as well as pixel-level representation of images provides important information that can be used to robustly estimate the number of panels in the image. Specifically, our approach starts by automatically extracting figure-caption pairs from full-text articles in PDF files. We then analyze the captions to identify the numbering style (e.g., A), B), C)..., I, II, III, etc.) and number of panels in the figures. Similarly, our solution analyzes the text embedded in the figures to locate the position and number of the panel labels in the figure. We then applied several image processing techniques using pixel-level representations to compute the optimum figure partition, and generates a set of minimum-area rectangles bounding the objects in inside the figure. Finally, we estimate the panel layout and use the layout to optimally partition the figure.

Our framework can be applied to a broad range of biomedical data mining tasks, although in our current work we focus on PPI mining from literature, a critical contribution to PPI assertions [31]. Specifically, we have used a set of 2, 843 articles from the MINT database, which is a manually curated database of Biomedical articles describing experimental evidence of interaction between proteins. On average, this articles contains 6 figures, each figure containing 3 panels (subfigures). Experimental results show that our approach achieved an accuracy of 96.64% in panel segmentation, greatly surpassing pure caption-based and pure figure-based approaches.

Our future work will focus on improving the performance of our system, evaluating the performance in other data mining tasks, and making it as a general curation tool. Specifically, our approach to extract figure-caption pairs from biomedical publications assumes that figures are described as image operators in the PDF, that is, each subfigure is an image. This assumption is not always valid, especially for information graphs such as bar-charts and line-charts, in which a figure can be described using a number of line primitives rather than a single image. As a result, a single subfigure can lead to a large number of primitives. We plan to develop an approach to identify a group of primitives using machine learning and other techniques, and then develop case-specific algorithms to handle each individual type of information graphs.

In our panel segmentation solution, the evaluation of the experimental results shows that our solution is sensitive to errors generated by the OCR tool (e.g., noisy characters). This is because our solution assumes that the text recognized from the figures is complete and correct. As shown in our results, this assumptions fails in figures with complex background, and in regions with low contrast. We plan to resolve this issue by pre-processing the OCR data to improve the noisy text using Natural Language Processing (NLP) methods, such as statistical language modeling.

## Competing interests

The authors declare that they have no competing interests.

## Authors' contributions

COT implemented the text parser to identify the panel labels and subcaptions. LDL implemented the imageprocessing and ML algorithms and wrote the first draft of the manuscript in collaboration with MT, JY, COT and CNA. KVS, CW, HG gave substantial contributions to develop our proposed pipeline; all authors critically revised the manuscript and approved its final version.

## Declarations

The publication costs of this article were funded by the NSF (National Science Foundation) 2013, University of Delaware, awarded to Jingyi Yu. This manuscript is an expansion of the article [32] published as part of IEEE International Conference on Bioinformatics and Biomedicine (BIBM) 2012.

This article has been published as part of *BMC Systems Biology *Volume 7 Supplement 4, 2013: Selected articles from the IEEE International Conference on Bioinformatics and Biomedicine 2012: Systems Biology. The full contents of the supplement are available online at http://www.biomedcentral.com/bmcsystbiol/supplements/7/S4.
